# Temporal Trends in the Association between Participation in Physical Education and Physical Activity among U.S. High School Students, 2011–2017

**DOI:** 10.3390/ijerph17072357

**Published:** 2020-03-31

**Authors:** Seungho Ryu, Paul Loprinzi, Heontae Kim, Minsoo Kang

**Affiliations:** 1 Department of Health, Exercise Science and Recreation Management, The University of Mississippi, University, MS 38677, USA; sryu2@go.olemiss.edu (S.R.); pdloprin@olemiss.edu (P.L.); 2 School of Applied Sciences, The University of Mississippi, University, MS 38677, USA; hkim35@olemiss.edu

**Keywords:** physical activity, physical education, trend

## Abstract

The purpose of this study was to assess trends in physical education and physical activity among U.S. high school students from 2011 to 2017, respectively, and to evaluate temporal trends in the relationship between physical education class participation and physical activity levels. Data from a total of 51,616 high school students who participated in the Youth Risk Behavior Surveillance System (YRBSS) from 2011 to 2017 were analyzed for this study. Physical education was measured as follows: In an average week when you are in school, on how many days do you go to physical education classes? Physical activity was measured as follows: During the past 7 days, on how many days were you physically active for a total of at least 60 min per day? Meeting physical activity guideline was defined as engaging in 7 days per week of at least 60 min per day. Across the 2011–2017 YRBSS, there were no linear (*p* = 0.44)/quadratic trends (*p* = 0.37) in physical education and linear (*p* = 0.27)/quadratic trends (*p* = 0.25) in physical activity, respectively. Regarding the trends in the association between physical education and physical activity, there were no statistically significant linear (unstandardized regression coefficient (b) = 0.013, *p* = 0.43) or quadratic (*p* = 0.75) trends; however, at the sample level, there was a slight increase in the odds ratio effect sizes from 2011 to 2017 (ES = 1.80–1.98). The pooled association between physical education participation and physical activity was statistically significant, OR = 1.88, 95% CI = 1.75–2.03, *p* < 0.001. That is, on average across the four YRBSS cycles, students who participated in physical education had 1.88 times higher odds of meeting physical activity guideline when compared to students who did not participate in physical education. The trends of physical education and physical activity did not change between 2011 and 2017. Overall, we observed a significant relationship between participation in physical education and physical activity. There was a non-significant yet stable trend of association between participation in physical education and physical activity over time.

## 1. Introduction

According to the National Association of State Boards of Education, physical education is defined as “focused on teaching school-aged children the science and methods of physically active, healthful living” [1]. Participation in physical education is associated with numerous benefits [2,3], including favorable social [4,5,6], affective [7,8], and cognition [9,10] outcomes. Participating in physical education is also positively related to increased academic performance [11]. 

Physical activity is defined as “any bodily movement produced by skeletal muscles that result in energy expenditure” [12]. Physical activity is associated with various positive effects, such as reductions in cardiovascular disease [13], obesity [14], and metabolic syndrome [15]. Especially noteworthy is the importance of physical activity during adolescents, which may impact health outcomes during adulthood [16,17].

Physical education may play an important role in promoting youth physical activity [18] because participation in physical education may affect the motivation of students to get involved in physical activity [19]. This positive motivation of students in physical education could affect children to start physically active lifestyles as adults [18]. Demonstrating an association between physical education and physical activity levels will underscore the importance of physical education. Further, physical education curriculum and physical activity levels have changed over the years [20,21], and thus, such changes may have altered the association between physical education participation and physical activity levels. A comprehensive understanding of participating in physical education and physical activity-related secular trends is necessary because it is an indicator of how the relationship between physical education and physical activity changes over time in public health. According to previous studies, the trends of physical education and of physical activity have been studied only until the late 2000s [22,23,24,25]. As such, follow-up work using more recent data is needed.

Therefore, the purposes of this study were to assess trends in physical education and in physical activity among US high school students from 2011 to 2017, respectively, and to evaluate secular trends in the relationship between physical education participation and physical activity levels. In addition, we evaluated the pooled association between physical education participation and physical activity levels across the years of 2011 to 2017. These research questions have important public health implications. 

## 2. Materials and Methods 

### 2.1. Study Design and Participants

The Youth Risk Behavior Surveillance System (YRBSS) is an ongoing survey as well as a nationally representative, cross-sectional, and school-based survey conducted by the Center for Disease Control and Prevention (CDC) since 1991 for focusing exclusively on health-risk behaviors. The survey uses a three-stage cluster sample design; multistage, clustered probability sampling design to obtain a sample of students from grades 9–12 who attend public and private schools in the 50 states and the District of Columbia. Additional details of the sampling strategy and psychometric properties of the 2015 YRBSS are reported elsewhere [26]. Participation in the YRBSS is entirely voluntary and anonymous and conducted in accordance with local requirements for parental permission prior to data collection. To evaluate trends in physical education, physical activity, and the association between participating physical education classes and meeting physical activity guidelines, a total of 51,616 participant’s data from the 2011–2017 YRBSS cycles were utilized. Missing data were not imputed. CDC’s institutional review board has approved the national YRBSS.

### 2.2. Physical Education Class

Physical education was measured as follows: In an average week when you are in school, on how many days do you go to physical education classes? Response options included “0 days”; “1 day”; “2 days”; “3 days”; “4 days”; “5 days”. For the analysis, physical education is defined into two groups: (1) no participating physical education class per week; (2) participating in at least 1 day per week of physical education classes. 

### 2.3. Physical Activity 

The outcome variable, physical activity, was measured as follows: During the past 7 days, on how many days were you physically active for a total of at least 60 min per day? Response options included “0 days”; “1 day”; “2 days”; “3 days”; “4 days”; “5 days”; “6 days”; “7 days”. Meeting physical activity guidelines was defined as engaging in 7 days per week of at least 60 min per day [27]. 

### 2.4. Body Mass Index (BMI)

BMI was categorized into four levels for assessing obesity status: (1) obese (i.e., BMI ≥ 95th percentile by age and gender), (2) overweight (i.e., 85th percentile ≤ BMI < 95th percentile by age and gender), (3) normal weight (i.e., BMI < 85th percentile by age and gender), and (4) underweight (i.e., BMI < 5th percentile by age and gender).

### 2.5. Statistical Analysis

All analyses conducted in Stata^®^ (v. 12, StataCorp, College Station, TX, USA) took into account the complex sampling design of the survey; sample weights, primary sampling units, and clustering parameters were used to adjust for non-response, non-compliance, and to render nationally representative estimates. 

Multiple logistic regression was used to evaluate the association between participating in physical education and meeting physical activity guidelines (outcome variable) for each of four YRBSS cycles (2011–2017). In each model, covariates included age, gender, race-ethnicity, and BMI. 

Comprehensive Meta-Analysis (CMA, Biostat Inc., Englewood, NJ, USA) software was used to evaluate the trend in the effect size (association) between participating in physical education and meeting physical activity guidelines. Specifically, we used a meta-regression to examine the trend of the relationship between the observed odd ratio effect sizes and the YRBSS cycles (year) using a random-effects model (under the assumption of between-study heterogeneity). The degree of heterogeneity of the effect sizes was evaluated with the Cochran’s Q-statistic and the proportion of variation attributable to between-study heterogeneity was evaluated with I^2^ index. 

For each trend analysis, tests for linear and quadratic trends were conducted using orthogonal polynomial coefficients. A significance level was set at 0.05. 

## 3. Results

Table 1 shows the weighted demographic characteristics. Overall, the demographic characteristics, including gender, race-ethnicity, and BMI, were similar across the YRBSS cycles. The weighted gender proportions range was similar to both males (48.71%–51.02%) and females (48.98%–51.29%) across the respective cycles. The range of weighted race/ethnicity proportions depends on the respective cycles: White (54.90%–58.82%), Black or African American (12.27%–13.47%), Hispanic/Latino (19.33%–22.43%), and other (8.73%–10.40%) proportions were similar. The weighted BMI proportions ranges were stable across the YRBSS cycles: obese (13.76%–15.65%), overweight (16.65%–18.06%), and normal (64.58%–67.44%). 

**Figure 1:**
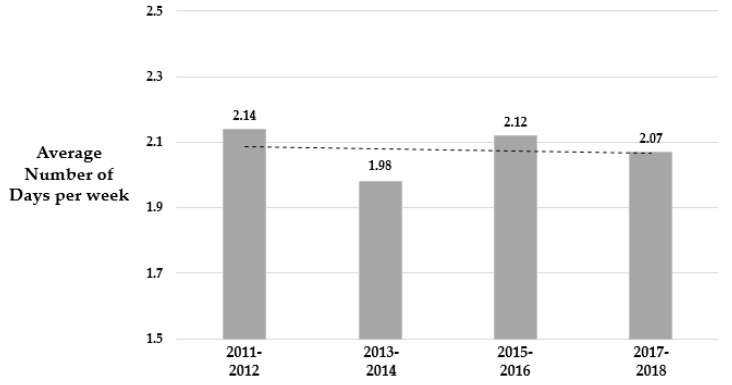
Weighted mean number of participation in physical education for the overall sample from 2011 to 2017 (*N* = 51,616).

Table 2 indicates the weighted mean number of days per week in physical education class for the overall sample and population subgroups across the measured YRBSS cycles from 2011 to 2017, respectively. For the overall sample, there were no linear and quadratic trends (P_linear_ = 0.44 and P_quadratic_ = 0.37). Sex, race-ethnicity, and BMI have been shown to have similar results. However, there was a slight decrease in the number of days per week in physical education class across the YRBSS cycles (Figure 1).

**Figure 2:**
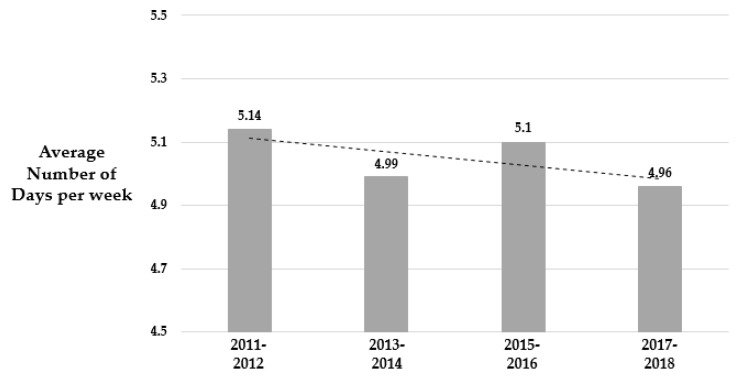
Weighted mean number of days physically active days for the overall sample from 2011 to 2017 (*N* = 51,616).

Table 3 presents the trends in the weighted average number of days per week in at least 60 min of physical activity for the overall sample and subgroups across the evaluated YRBSS cycles from 2011 to 2017, respectively. For the overall sample, there were no linear and quadratic trends (P_linear_ = 0.27 and P_quadratic_ = 0.25). However, there was a slight decrease in the number of days per week in physical activity across the YRBSS cycles (Figure 2). A similar result was found in gender and race-ethnicity. We observed a negative linear trend of physical activity in the obese group (P_linear_ = 0.03); however, there were no statistically significant trends of physical activity in the overweight and normal groups. According to our results, the average number of days per week in at least 60 min of physical activity was lower in the obese group than in the overweight and normal groups.

**Figure 3:**
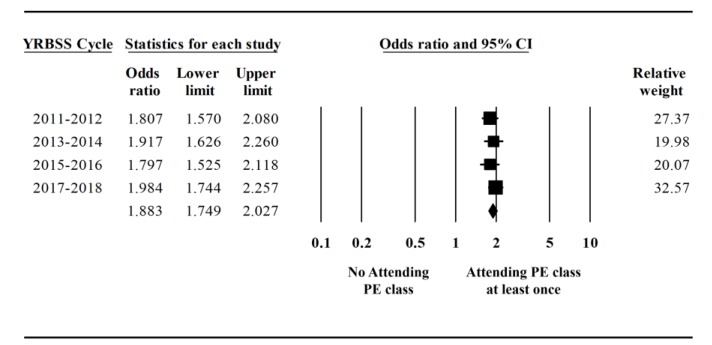
Odds of meeting physical activity guidelines based on participating physical education classes.

**Figure 4:**
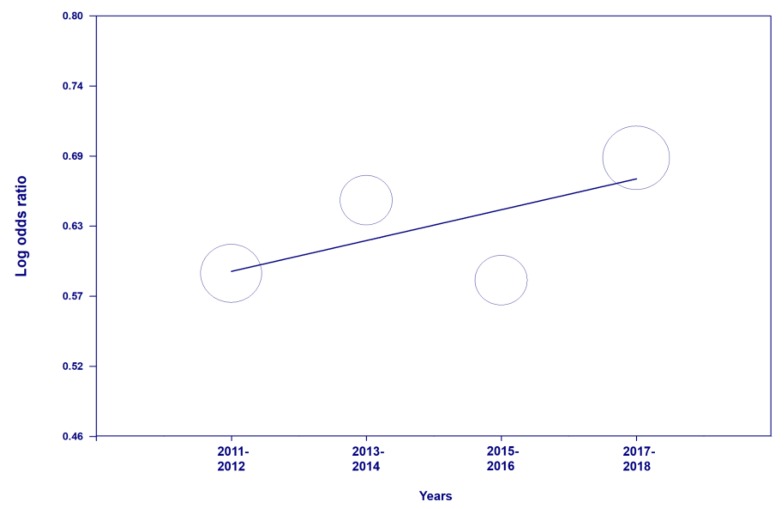
Trend in the association between participating physical education classes and meeting physical activity guidelines across the 4 cycles.

Figure 3 displays the results for the association between participating in physical education class and meeting physical activity guideline. Across the years of 2011 to 2017, the association between participating in physical education and meeting physical activity guideline was statistically significant (OR = 1.88, 95% CI = 1.75–2.03, *p* < 0.001). That is, participating in at least one physical education class per week was associated with an 88% increased odds of meeting physical activity guidelines. Regarding the trend in the association between participating physical education class and meeting physical activity guideline across the four YRBSS cycles, there was no statistically significant trend (b = 0.013, *p* = 0.43); however, there was a slight increase in the odds ratio across the YRBSS cycles (Figure 4). There was no evidence of heterogeneity (Q(7) = 1.32, *p* = 0.73, I^2^ = 0%).

## 4. Discussion

Previous studies have demonstrated a relationship between physical education or physical activity and diverse health outcomes, such as physical health [28,29,30], cognitive/academic development [31,32,33], mental health [30,34], and crime reduction [35,36]. However, to date, no research has evaluated the trend of the relationship between participation in physical education and meeting physical activity guidelines. Therefore, the purpose of this study was to assess trends in the association between physical education participation and meeting physical activity guidelines among the American high school student population. An additional aim of the study was to evaluate trends in physical education and physical activity among U.S. high school students, respectively. In the results of physical activity trends, between 2011 and 2017, there was a negative linear trend in the obese group, whereas there was not any trend in the physical education. Further, our results indicated that there were no linear and quadratic trends in the association between participation in physical education and meeting physical activity guidelines among the broader high school student population between 2011 and 2017. However, we found that students with at least one day of participation in physical education were more likely to meet physical activity guidelines within each of the YRBSS cycles and across the four cycles as well. 

In the trend of physical education, it is difficult to directly compare our results due to the lack of previous research on trends of participation in physical education. However, according to Lowry et al., the trend of participation in physical education class in the high school student population did not change between 1991 (48.9%) and 1997 (48.8%) [22]. Further, Lee et al. concluded that since 2000, the prevalence of daily physical education has not decreased significantly, but it has not increased either [23]. To sum up the previous studies and our results, the trend of participation in physical education class did not change from 1991 to 2017. The reason for the unchanging trend is probably that most states and districts would have required physical education at all school levels, and only 4.8% of high schools did not have a physical education requirement [23]. However, more than 20% of high schools requiring physical education have granted exemptions for other school-based activities such as bands, choruses, and school-based sports [37]. Allowing the exemption reduces the importance and support of all students’ awareness of participation in physical education. Therefore, states and school districts should minimize the reasons for which students can be exempted from participating in physical education to improve the quality and number of those participating in physical education. 

In the trend of physical activity, although there was a slight increase in physical activity in youths aged 11–15 years who met the physical activity guidelines between 2002 and 2010 in 32 European and North American countries, the United States decreased in physical activity [24]. The National Representative YRBSS of the United States found a decreasing pattern for youth in physical activity from 2003 to 2006 [25]. Overall, previous studies have shown that youth’s physical activity declined from 2002 to 2010 in the United States. However, our results indicated the trend of overall sample physical activity shows a stable pattern from 2011 to 2017. This result indicates physical activity continued to decline until 2011 but continued steadily until 2017 without any increase or decrease movement in physical activity. In the obese group, the trend of physical activity decreased between 2011 and 2017. This may be due to the fact that adolescents in the obese group have increased TV viewing time. According to a previous study, adolescents who watch more than 2 h of television/video every day are almost twice as likely to be obese compared to their normal-weight peers [38]. TV watching and media use may reduce the chances of physical activity and encourage the intake of unhealthy food via advertising of fast food shown while viewing [39,40,41]. 

According to previous studies of association between participating in physical education and meeting physical activity, 30 min of physical education immediately contributes to daily physical activity in children [42]. That is, moderate and more active children accumulated about 1100 to 2500 more steps on a day with a physical education class compared to a day without a physical education class [42]. Kang and Brinthaupt examined the effect of pedometer-based intervention in school physical education classes and showed an average of 1000 steps increased from the baseline to post-intervention [43]. Furthermore, Cawley et al. suggested that longer physical education time increased the number of days of moderate to vigorous physical activity by at least 20 min [44]. Our finding that high school students who participated in at least one physical education class were more likely to meet physical activity guidelines is supported by previous studies.

Our study has some limitations. First, because of the cross-sectional nature of the YRBSS data, the cause and effect relationship between participating in physical education and physical activity could not be determined. Second, YRBSS data are based on self-report, which could be subject to biases in recall and social desirability.

## 5. Conclusions

The overall association between participating physical education and meeting physical activity guidelines of the US high school population demonstrated that there was a stable trend from 2011 to 2017. Although the trend was not statistically significant, it has slightly increased over time. Further, the trends of participation in physical education and physical activity did not change. For a better understanding of these trends, monitoring may be needed to see such changes, and this will provide useful information about when resources should be allocated to develop, implement, and evaluate physical education and physical activity in school.

## Figures and Tables

**Table 1:** Weighted prevalence of demographic characteristics across the evaluated cycles (2011–2017 YRBSS; *N* = 51616) ^a^.

Characteristic	2011 (*n* = 13,516)	2013 (*n* = 12,020)	2015 (*n* = 13,643)	2017 (*n* = 12,437)
**Gender (%)**				
Male	50.87 (49.06–52.68)	49.36 (48.11–50.60)	51.02 (47.77–54.26)	48.71 (46.43–51.00)
Female	49.13 (47.32–50.94)	50.64 (49.40–51.89)	48.98 (45.74–52.23)	51.29 (49.00–53.57)
**Race/Ethnicity (%)**				
White	58.82 (51.61–66.04)	57.21 (49.11–65.31)	56.14 (48.59–63.70)	54.90 (48.56–61.24)
Black or African American	13.12 (9.54–16.69)	13.47 (8.88–18.06)	12.82 (9.21–16.44)	12.27 (9.42–15.12)
Hispanic/Latino	19.33 (13.95–24.72)	20.56 (15.24–25.87)	21.58 (15.61–27.55)	22.43 (17.01–27.87)
Other	8.73 (6.69–10.76)	8.76 (6.79–10.74)	9.45 (7.33–11.57)	10.40 (8.76–12.03)
**BMI (%)**				
Obese	13.76 (12.34–15.17)	14.67 (13.33–16.00)	14.82 (13.19–16.45)	15.65 (14.40–16.90)
Overweight	16.65 (15.70–17.59)	18.06 (16.98–19.15)	17.73 (16.76–18.70)	17.28 (16.45–18.11)
Normal	67.44 (65.69–69.18)	64.76 (62.71–66.81)	65.19 (63.11–67.27)	64.58 (62.95–66.22)

^a^ YRBSS = Youth Risk Behavior Surveillance System.

**Table 2:** Weighted mean number of days per week youth engaged in physical education classes (2011–2017 YRBSS; *N* = 51,616) ^a^.

Sample	2011 (*n* = 13,516)	2013 (*n* = 12,020)	2015 (*n* = 13,643)	2017 (*n* = 12,437)	P-Trend_linear_^b^	P-Trend_quadratic_^c^
**Overall Sample**	2.14 (1.89–2.39)	1.98 (1.75–2.22)	2.12 (1.86–2.38)	2.07 (1.83–2.31)	0.44	0.37
Gender						
Male	2.35 (2.12–2.58)	2.26 (2.05–2.46)	2.30 (2.01–2.59)	2.32 (2.06–2.58)	0.24	0.45
Female	1.91 (1.61–2.21)	1.72 (1.44–2.00)	1.93 (1.67–2.20)	1.84 (1.61–2.07)	0.27	0.26
**Race/Ethnicity**						
White	2.18 (1.85–2.52)	1.80 (1.57–2.02)	1.96 (1.67–2.25)	1.92 (1.66–2.18)	0.38	0.15
Black or African American	1.96 (1.69–2.22)	2.00 (1.63–2.37)	2.39 (2.10–2.68)	2.11 (1.89–2.34)	0.10	0.30
Hispanic/Latino	2.16 (1.93–2.40)	2.47 (2.17–2.75)	2.37 (2.05–2.69)	2.40 (2.06–2.74)	0.69	0.44
Other	2.04 (1.78–2.29)	2.09 (1.80–2.37)	2.12 (1.70–2.55)	2.13 (1.83–2.44)	0.88	0.92
**BMI**						
Obese	2.05 (1.77–2.34)	2.03 (1.75–2.30)	2.14 (1.87–2.41)	1.98 (1.72–2.25)	0.57	0.75
Overweight	2.15 (1.94–2.36)	1.99 (1.74–2.24)	2.24 (1.98–2.51)	2.13 (1.88–2.37)	0.18	0.18
Normal	2.16 (1.89–2.43)	1.97 (1.74–2.21)	2.10 (1.83–2.37)	2.09 (1.84–2.34)	0.49	0.34

^a^ YRBSS = Youth Risk Behavior Surveillance System. ^b^ Tests for linear trends were conducted using linear-specific orthogonal polynomial coefficients. ^c^ Tests for quadratic trends were conducted using quadratic-specific orthogonal polynomial coefficients.

**Table 3:** Weighted mean number of days per week youth engaged in at least 60 m/d of physical activity (2011–2017 YRBSS; *N* = 51,616) ^a^.

Sample	2011 (*n* = 13,516)	2013 (*n* = 12,020)	2015 (*n* = 13,643)	2017 (*n* = 12,437)	P-Trend_linear_^b^	P-Trend_quadratic_^c^
**Overall Sample**	5.14 (5.03–5.26)	4.99 (4.88–5.10)	5.10 (4.95–5.24)	4.96 (4.79–5.13)	0.27	0.25
**Gender**						
Male	5.74 (5.63–5.86)	5.57 (5.49–5.66)	5.62 (5.45–5.79)	5.56 (5.44–5.68)	0.63	0.33
Female	4.52 (4.39–4.66)	4.42 (4.28–4.57)	4.55 (4.36–4.74)	4.39 (4.17–4.61)	0.31	0.40
**Race/Ethnicity**						
White	5.34 (5.20–5.49)	5.17 (5.02–5.31)	5.32 (5.13–5.52)	5.07 (4.84–5.31)	0.21	0.28
Black or African American	4.78 (4.60–4.96)	4.58 (4.42–4.74)	4.71 (4.42–4.99)	4.64 (4.40–4.87)	0.47	0.27
Hispanic/Latino	4.89 (4.76–5.01)	4.84 (4.64–5.06)	4.85 (4.69–5.00)	4.89 (4.70–5.09)	0.93	0.74
Other	4.91 (4.70–5.13)	4.85 (4.64–5.05)	4.84 (4.58–5.09)	4.86 (4.65–5.08)	0.96	0.90
**BMI**						
Obese	4.73 (4.57–4.90)	4.53 (4.32–4.74)	4.83 (4.67–4.98)	4.61 (4.42–4.81)	*p* = 0.03	0.07
Overweight	5.14 (5.00–5.29)	5.00 (4.83–5.18)	5.00 (4.79–5.21)	4.91 (4.72–5.10)	0.98	0.91
Normal	5.24 (5.12–5.37)	5.11 (5.01–5.21)	5.20 (5.04–5.37)	5.07 (4.87–5.27)	0.34	0.30

^a^ YRBSS = Youth Risk Behavior Surveillance System. ^b^ Tests for linear trends were conducted using linear-specific orthogonal polynomial coefficients. ^c^ Tests for quadratic trends were conducted using quadratic-specific orthogonal polynomial coefficients.
